# Patient-reported performance status and postoperative complications in elective colorectal cancer surgery

**DOI:** 10.1007/s00384-024-04761-1

**Published:** 2024-11-21

**Authors:** Helin Yikilmaz Pardes, Niclas Dohrn, Troels Gammeltoft Dolin, Ismail Gögenur, Mads Falk Klein

**Affiliations:** 1 Department of Surgery, Copenhagen University Hospital – Herlev and Gentofte, Borgmester Ib Juuls Vej 1, 2730 Herlev, Denmark; 2 Department of Surgery, Zealand University Hospital, University of Copenhagen, Lykkebækvej 1, 4600 Køge, Denmark; 3 Department of Medicine, Copenhagen University Hospital – Herlev and Gentofte, Borgmester Ib Juuls Vej 1, 2730 Herlev, Denmark

**Keywords:** Performance status, Postoperative complications, Patient-reported outcome measures, Colorectal cancer surgery

## Abstract

**Purpose:**

The purpose of this study was to evaluate the concordance between patient-reported performance status (prPS) and surgeon-reported performance status (srPS), and to assess the correlation between srPS and prPS and postoperative complications following elective colorectal cancer surgery. Not all patients are deemed suitable for undergoing a surgical procedure. We aimed to assess whether prPS can aid the surgeons’ decision-making prior to surgery.

**Methods:**

In this retrospective study, 524 patients undergoing colorectal cancer surgery were included. prPS were collected via questionnaires, while 30-day postoperative complications were obtained from the Danish Colorectal Cancer Group (DCCG) database. To evaluate the agreement between prPS and srPS, linearly weighted kappa statistics were applied. Rank-biserial correlation analysis was used to calculate the correlation between prPS and srPS with postoperative complications.

**Results:**

In total, there was an approximate 71% concordance between the assessments. Around 17% of the patients rated themselves with a higher PS status than the surgeons, while 13% of the patients rated themselves with a lower PS. Overall postoperative complications, minor surgical complications, and medical complications were all significantly correlated to both srPS and prPS, while only srPS was correlated with major surgical complications. Neither srPS nor prPS were correlated with overall surgical complications (major and minor collapsed).

**Conclusion:**

The agreement between prPS and srPS is poor and in nearly one-third of the cases, disagreement occurs. Overall, both prPS and srPS were correlated to postoperative complications, with srPS demonstrated a slightly higher correlation.

## Introduction

Surgery remains the mainstay of the treatment of localized colorectal cancer (CRC). However, not all patients are suitable for a surgical procedure, and there are multiple available methods for predicting outcomes to surgery in different high-risk populations. Several preoperative risk prediction models have previously been proposed, such as the Physiological and Operative Scoring System for enumeration of Morbidity and Mortality (POSSUM) [1], Estimation of Physiologic Ability and Surgical Stress Score (E-PASS) [2], National Surgical Quality Improvement Program Risk Calculator (NSQIP) [3], and comprehensive geriatric assessment (CGA) [4]. However, these models are complex, time-consuming, and not widely implemented in treatment algorithms.

World Health Organization performance status (PS) [5] is a worldwide known measure used by healthcare professionals to estimate a patient’s general health status [6, 7]. In CRC surgery, surgeons use PS, together with tumor characteristics, patients’ comorbidity, and their clinical judgment, for risk stratification and to decide whether the patient is suitable for surgery [8]. Patients with a high PS are at increased risk of adverse short- and long-term outcomes after undergoing surgery for CRC, and the 30- and 90-day mortalities are higher for patients with a PS 1 or above compared with PS 0 [9]. For older patients with cancer, PS is a factor that influences 90-day postoperative mortality [10], with a significantly higher 90-day postoperative mortality among patients with PS 4 (HR 16.3, 95% CI 8.27–32.1) compared to patients with PS 2 (HR 1.09, 95% CI 0.81–1.47) [10].

In recent years, patient-reported outcome measures (PROMs) are receiving significantly more attention. PROMs are considered as important outcome measures when it comes to evaluating the effect of treatment and the patients’ well-being compared to the outcomes assessed by the physician [11]. In clinical cancer trials, PROMs, mainly quality of life, have shown to have a prognostic significance for survival among patients diagnosed with cancer [12]. To our knowledge, patient-reported performance status (prPS) prior to CRC surgery has not been investigated previously. It can be argued that the surgeon-reported performance status (srPS) is based on a short evaluation and impression of the patient, and therefore can be deficient compared to the PS reported by the patient. Nevertheless, while it is plausible to assume that patients may be better at evaluating their own physical ability, we do not know if prPS has any predictive value for postoperative complications. This study aimed to compare the agreement between prPS and srPS and investigate whether prPS is correlated with postoperative complications after elective CRC surgery.

## Materials and methods

### Study design and data collection

This study is based on patients included in the REBECCA study (“Biomarkers in patients with colorectal cancer – can they provide new information on the diagnosis, treatment efficacy, adverse effects and prognosis?”) [13], and additional patient data and outcomes were collected from the Danish Colorectal Cancer Group (DCCG) database [14].

REBECCA is an exploratory observational study with prospective collection of clinical data together with a health-related questionnaires on physical activity, diet, weight loss, and other health and lifestyle-related issues, prior to either surgical or oncological treatment in patients with all UICC stages (I–IV) of CRC and has been previously been reported [13]. Patients with verified or suspected CRC undergoing resection with curative intent, above 18 years of age, and capable of giving informed consent, were applicable for inclusion. The REBECCA study was approved by the Regional Ethical Committee (VEK j.nr. H-2–2013-078) and the Danish Data Protection Agency (j. No. HEH-2014–044, I-suite No. 02771 and PACTIUS P-2019–614).

The DCCG database prospectively collects nationwide data and has a high degree of data completeness with more than 99% of all patients above 18 years of age diagnosed with a primary colorectal malignant lesion in Denmark included in the database [14]. The data encompass pre-, intra-, and postoperative variables and in a recent validation study the database showed an overall high completeness, conformity, and accuracy of data [15].

From the REBECCA database, we selected all patients who had undergone surgical resection for CRC, and who had completed the health-related questionnaires, from the start of the database in July 2014 through June 2020. Patient, treatment, and tumor characteristics were collected from the DCCG database. In case of missing data on the variables of postoperative complications or srPS, medical records were reviewed by the author (HP).

### Patient- and surgeon-reported performance status

Prior to surgery, the patients were asked to fill out the health-related questionnaire, in which they reported their own prPS by setting a tick beside the statement that most accurately reflected their functional status. The definitions of PS are shown in Table 1. The srPS were retrieved from the DCCG database based on a consultant surgeon evaluation.

**Table 1:** Definition of performance status

GRADE	WHO PERFORMANCE STATUS
0	Fully active, able to carry on all pre-disease performance without restriction
1	Restricted in physically strenuous activity but ambulatory and able to carry out work of a light or sedentary nature, e.g., light housework, office work
2	Ambulatory and capable of all self-care but unable to carry out any work activities; up and about more than 50% of waking hours
3	Capable of only limited self-care; confines to bed or chair more than 50% of waking hours
4	Completely disabled; cannot carry on any self-care; totally confined to bed or chair
5	Dead

### Postoperative complications

We retrieved data on 30-day postoperative complications from the DCCG database on the selected patients. In the database, the most frequent organ-related surgical and medical postoperative complications are graded individually according to the Clavien-Dindo classification (CD) [16]. This was used to assess the overall rate of patients with postoperative complications, and additionally, the specific rates of surgical, medical, major, and minor complications. Major complications were defined as CD > 3a, whereas minor complications were defined as CD < 3b.

We planned to compare the prPS to the srPS and to investigate the association of prPS with postoperative complications. In effect, the primary outcome of interest was the agreement between the prPS and the srPS, and the secondary outcome was the correlations of prPS and srPS with 30-day postoperative complication rates.

### Statistical methods

Linearly weighted kappa statistics was used to evaluate the agreement between prPS and srPS [17].

Rank-biserial correlation analysis [18] was used to calculate prPS and srPS correlation with postoperative complications. The correlation analysis was performed on overall, major, minor, surgical, and medical postoperative complications individually, and reported as an estimate together with a 95% confidence interval.

Two-sided statistics with a significance level of 0.05 were applied. For all the statistical analyses, R-studio (RStudio, PBC, Boston, MA, USA) was used, and the study was reported according to the Strengthening the Reporting of Observational Studies in Epidemiology (STROBE) statement.

## Results

In the selected period, 817 patients were included in REBECCA study. Preoperative questionnaires were completed for 542 patients. Patients who were diagnosed with other pathology than CRC (e.g., anal cancer) were excluded (*n* = 18). Patients who received neoadjuvant treatment were also excluded. In total, 524 patients were included in the final analysis.

Demographic, clinical characteristics of the patients, and postoperative complications are shown in Table 2.

**Table 2:** Patient clinicopathological demographics

*Variables*			*Missing*
*n*		524	
*Gender (%)*	Male	297 (56.7)	0.0
	Female	227 (43.3)	
*Age (median [IQR])*		69.00 [62.00, 75.00]	0.0
*BMI (median [IQR])*		25.26 [23.13, 27.77]	8.0
*ASA (%)*	I	179 (34.7)	1.5
	II	283 (54.8)	
	III	54 (10.5)	
*Surgeon-reported PS (%)*	PS 0	412 (80.2)	1.9
	PS 1	69 (13.4)	
	PS 2	29 (5.6)	
	PS 3	4 (0.8)	
	PS 4	0 (0.0)	
*Patient-reported PS (%)*	PS 0	348 (72.8)	8.8
	PS 1	104 (21.8)	
	PS 2	22 (4.6)	
	PS 3	3 (0.6)	
	PS 4	1 (0.2)	
*Cancer location (%)*	Colonic	370 (70.6)	0.0
	Rectal	154 (29.4)	
*Procedure (%)*	Right colectomy	167 (31.9)	0.0
	Left colectomy	41 (7.8)	
	Sigmoidectomy	127 (24.2)	
	Colon resection, unspecified	11 (2.1)	
	Total colectomy	7 (1.3)	
	Low anterior rectal resection	126 (24.0)	
	Abdominoperineal resection	42 (8.0)	
	Proctocolectomy	3 (0.6)	
*Surgical approach (%)*	Open	40 (7.6)	0.0
	Laparoscopic	260 (49.6)	
	Robotic	224 (42.7)	
*Conversion to open (%)*	Yes	57 (11.0)	1.5
	No	459 (89.0)	
*Clinical T category (%)*	T0	2 (0.7)	48.7
	T1	41 (15.2)	
	T2	111 (41.3)	
	T3	94 (34.9)	
	T4	21 (7.8)	
*Clinical N category (%)*	N0	161 (55.5)	44.7
	N1	72 (24.8)	
	N2	30 (10.3)	
	Nx	27 (9.3)	
*Clinical M category (%)*	cM0	475 (94.2)	3.8
	cM1	25 (5.0)	
	cMx	4 (0.8)	
*Pathological T category (%)*	pT1	71 (14.3)	5.2
	pT2	104 (20.9)	
	pT3	256 (51.5)	
	pT4	60 (12.1)	
	pT0	6 (1.2)	
*Pathological N category (%)*	pN0	322 (64.8)	5.2
	pN1	119 (23.9)	
	pN2	56 (11.3)	
*Pathological UICC (%)*	I	134 (28.8)	11.3
	II	150 (32.3)	
	III	151 (32.5)	
	IV	30 (6.5)	
*Overall complications (%)*	Yes	130 (24.8)	0.0
	No	394 (75.2)	
*Major complications (%)*	Yes	48 (9.2)	0.4
	No	474 (90.8)	
*Minor complications (%)*	Yes	100 (19.2)	0.4
	No	422 (80.8)	
*Surgical complications (%)*	Yes	80 (15.3)	0.0
	No	444 (84.7)	
*Medical complications (%)*	Yes	80 (15.3)	0.0
	No	444 (84.7)	
*30-day mortality (%)*	Yes	8 (1.5)	0.0
	No	516 (98.5)	

Values are given as absolute numbers (percentages) in parentheses unless otherwise is stated

Abbreviations: *BMI* body mass index, *ASA *American Society of Anesthesiology score, *PS* performance status, *UICC* Union of International Cancer Control

The median age was 69 years (IQR 62–75) and 56% of the patients were male. The majority of the patients (71%) had surgery for colonic cancer, while the remaining portion (29%) underwent surgery for rectal cancer. When assessing the tumor stage, 14.3% of the patients had T1 tumors and 20.9% had T2 tumors, while T3 and T4 tumors were observed in 51.5% and 12.1% of the cases, respectively. Approximately 7.6% of the patients underwent surgery with an open surgical approach and 49.6% underwent conventional laparoscopic surgery, while the remaining 42.7% underwent robot-assisted laparoscopic surgery. Of the minimally invasive cases, 11% of the cases were converted to open surgery.

In total, 24.8% of the patients experienced any kind of postoperative complication. Most frequently this was a minor complication (19.2%), whereas major complications were observed in 9.2% of the patients.

### Patient-reported and surgeon-reported performance status

The distribution of ratings in the two groups is presented in Table 3. The agreement between prPS and srPS is marked diagonally with italics. The overall agreement between prPS and srPS was poor/fair with a weighted kappa of 0.33 (*p* < 0.00001) [19, 20]. In total, there was approximately 71% agreement between PS assessments of the patients and the surgeons.

**Table 3:** Agreement table for patient-reported and surgeon-reported performance status

			Patient			
	Performance status	0	1	2	3	4
	0	*306 (65.4%)*	60 (12.8%)	10 (2.1%)	2 (0.4%)	0
Surgeon	1	36 (7.7%)	*19 (4.1%)*	5 (1.1%)	0	0
	2	1 (0.2%)	18 (3.8%)	*5 (1.1%)*	1 (0.2%)	1 (0.2%)
	3	0	2 (0.4%)	2 (0.4%)	*0*	0
	4	0	0	0	0	*0*

Italic indicates cases where agreement occurred between surgeon’s and patient’s assessment

The percentages indicate the proportion of the total cohort

A total of 60 (12.8%) of the patients rated themselves as PS 1. Upon assessment by the surgeon, all of these patients were determined to have a PS score of 0. Furthermore, approximately 17% of the patients rated themselves with a higher PS score compared to the evaluations made by the surgeons. Surgeons assessed 36 patients (7.7%) to have a PS of 1, whereas these patients rated themselves as PS 0. Approximately, 13%, rated themselves with a lower PS score compared to the assessments made by the surgeons.

### Correlation between performance status and postoperative complications

The results from the correlation analysis are shown in Table 4. Overall postoperative complications were correlated to both the srPS (*r*_rb_ = 0.142; 95% CI, 0.05–0.24) and the prPS (*r*_rb_ = 0.096; 95% CI, 0.009–0.183). The srPS was significantly correlated with major surgical complications (*r*_rb_ = 0.129; 95% CI, 0.051–0.206) whereas the prPS was not (*r*_rb_ = 0.035; 95% CI, − 0.023 to 0.944). Both prPS and srPS were significantly correlated with minor surgical complications and medical complications. Surgical complications (major and minor collapsed) were neither correlated to prPS nor srPS.

**Table 4:** Overview of the results of the Rank-Biseral correlation analysis

		Correlation coefficient (*r*_rb_)	95% CI
Overall complications	Surgeon assessed PS	**0.142**	**0.046 to 0.239**
	Patient-reported PS	**0.096**	**0.009 to 0.183**
Major surgical complications	Surgeon assessed PS	**0.129**	**0.051 to 0.206**
	Patient-reported PS	0.035	− 0.023 to 0.094
Minor surgical complications	Surgeon assessed PS	**0.097**	**0.006 to 0.189**
	Patient-reported PS	**0.111**	**0.029 to 0.193**
Surgical complication	Surgeon assessed PS	0.060	− 0.022 to 0.149
	Patient-reported PS	− 0.001	− 0.074 to 0.060
Medical complications	Surgeon assessed PS	**0.170**	**0.072 to 0.252**
	Patient-reported PS	**0.145**	**0.066 to 0.225**

Correlation coefficient ranges from −1 to 1, where values closer to 1 indicate stronger positive correlation. Bold indicates statistical significant correlation

Abbreviation: *PS*, performance status

## Discussion

We compared the agreement between prPS and the srPS and investigated which one had the best correlation with postoperative complications after elective CRC surgery. To our knowledge, this is the first study to investigate the significance of prPS among patients with CRC prior to surgery.

This study shows that despite the poor concordance between prPS and srPS, surgeons’ assessments remain the most strongly correlated to postoperative complications.

The overall agreement between prPS and srPS was poor/fair with a weighted kappa coefficient of 0.33. In 71% of the patients, there was an agreement between the two ratings.

Several studies have investigated the agreement between prPS and PS assessed by the physician — mainly oncologists, by using kappa weighted statistics [21–25]. A study from Japan investigated the prognostic value of performance status among 206 advanced non-small cell lung cancer patients assessed by patients themselves, oncologists, and nurses [21]. The agreement between oncologists and patients was worse than the agreement between oncologists and nurses (weighted kappa statistics were 0.53 and 0.63, respectively). In line with our results, they demonstrated that physicians give the lowest (i.e., healthiest) performance status assessment. In the study from Japan, they concluded that oncologists were better at evaluating PS than patients. From our results with a poor/fair agreement of 71% and a kappa of 0.33, we conclude that prPS and srPS in reality are different measures of PS and that their predictive values also could be different. In addition, asking patients to assess their own physical ability prior to surgery for CRC, and including them in the preoperative evaluation, would be of great importance in terms of patient involvement and patient-centered medicine. This is particularly relevant given that around 17% of patients perceived themselves to be in poorer health, and 13% to be in a better condition, when compared to the assessment performed by the surgeons. When evaluating prPS, it may be beneficial to consider the symptoms present at diagnosis. Symptoms such as obstruction or bleeding in CRC can lead to fatigue and reduced mobility and may adversely affect the prPS, potentially acting as confounding factors.

Even though patients are thought to be the best assessors of their own physical health condition and ability, they might not be able to express themselves because of their health status, cognitive impairment, or limited medical knowledge. The pessimistic prPS is also seen among patients with newly diagnosed small cell lung cancer and non-small cell lung cancer [23, 24]. The authors discuss whether a possible explanation could be underlying psychological comorbidity that is not assessed by the oncologists [24]. Our study had similar results with the majority of disagreement consisting of patients rating themselves worse than the surgeon. A study investigating the reasons behind the discrepancies in PS ratings found a statistically significant association between depression and patients who rated themselves as the most impaired [22]. The study also concluded that the major significant factor influencing the oncologists’ rating was the cancer stage. This suggests that the discrepancy in the two groups is to be expected, as the assessment of PS in each group is influenced by different factors.

The correlation analysis illustrated that srPS was correlated with overall, medical, major, and minor postoperative complications. prPS showed correlation with overall, medical, and minor postoperative complications. In general, the correlations seem to be more robust with srPS compared to prPS, but this was not statistically tested. By far, the strongest correlations for both measures of PS were with medical complications. These results are in line with nationwide data from the DCCG database, where medical complications occur more frequently in patients with higher PS [26].

Previous studies report that physician-rated PS is associated with postoperative complications and survival among patients with cancer [26–30]. In a study from the UK, poor PS among 10.991 patients who underwent surgical resection for non-small cell lung cancer was associated with early mortality [27]. In another study investigating the effect of comorbidity on survival among 3.152 patients resected for non-small cell lung cancer, increasing PS was independently associated with lower survival [28]. Survival within 1 month was lower among patients with PS 1 and 2 compared to patients with PS 0 [28]. For patients with bladder cancer, PS was an independent predictor of 5-year all-cause mortality in a study investigating 891 patients undergoing radical cystectomy [29]. A similar effect on survival was seen in a study where poor PS influenced overall survival among patients with gastric cancer after curative gastrectomy [30]. The DCCG annual report also demonstrates the effect of poor PS on postoperative complications, where patients in 2018 with poor PS were more disposed to get medical postoperative complications compared to patients with PS 0 (medical complications: PS 0, 4.3% vs PS 3, 20.2%) [26].

The strengths of this study lie in the robust sample size and the quality of the data concerning srPS and complications that was retrieved from the DCCG database. The database provides invaluable data for numerous scientific studies each year and has been validated where the completeness, and quality of the data was concluded to be high [15].

However, the study has important limitations. An important limitation to this study is the risk of patients not fully understanding the PS scale. The PS scale the patients were asked to use was not provided with lay language. In effect, the patients may not be equipped to use a PS scale designed for physicians. There is a possible risk of patients misinterpreting the descriptions of the scale in our study, and thereby both under- and overestimate their own physical ability. In a previous Canadian study, researchers used a layman-version of the PS scale when patients were asked to report their own PS. By doing so, their results were less affected by the patients’ socioeconomic background and literacy [25]. This could mean that our results might not accurately reflect the patients’ true assessment of their PS, as the understanding of the PS scale could be insufficient.

Another limitation to this study is that selection bias may be present as not all participants in the study completed the health questionnaire. A third limitation is that interobserver variability and assessment bias among the surgeons were not assessed in the study. However, an Australian study demonstrated that the interobserver variability among clinical oncologists is less using the WHO/Eastern Cooperative Oncology Group (ECOG) Performance Status scale, and that the scale therefore can be used with good interobserver reliability [31]. Lastly, incorporating Comprehensive Complication Index (CCI) evaluations alongside Clavien-Dindo scores in assessing postoperative complications could provide a clearer picture of the cumulative burden of complications. This approach allows for a more comprehensive assessment of patient outcomes by capturing both the severity and the total impact of all complications experienced.

The study holds substantial implications for the field of patient-centered care by shedding light on the dynamics between patients’ subjective assessments and clinical judgments.

Considering a patient’s self-assessment of their own function and health is highly valuable. It can complement the surgeon’s evaluation, leading to an enhanced overall holistic assessment and greater patient involvement throughout the treatment process. While other established screening tools like the fTRST (Functional Triage Risk Screening Tool) are valuable for efficiency and standardization, they may not fully reflect the subjective experiences of individuals. By combining the prPS and srPS, there is a potential for improved prognostic accuracy.

Future studies can build on these findings to enhance decision-making, refine assessment methods, and improve the integration of PROMs data into the clinical workflow.

In conclusion, agreement between prPS and srPS is poor and in nearly one-third of the cases disagreement occurs. It seems that the brief interaction between surgeon and patient prior to surgery does not hinder the surgeon’s capability to assess PS, as their correlation with postoperative complications appears to be slightly stronger than that of the patient.

## Data Availability

No datasets were generated or analyzed during the current study.
